# Do advanced glycation end-products play a role in malaria susceptibility?

**DOI:** 10.1051/parasite/2016015

**Published:** 2016-03-24

**Authors:** Karim Traoré, Charles Arama, Maurice Médebielle, Ogobara Doumbo, Stéphane Picot

**Affiliations:** 1 Malaria Research and Training Center MRTC-DEAP-FMPOS-UMI 3189, Université des Sciences, des Techniques et des Technologies de Bamako BP 1805 Bamako Mali; 2 Univ Lyon, Université Claude Bernard Lyon 1, Institut de Chimie, de Biologie Moléculaire et Supramoléculaire ICBMS-UMR5246, CNRS-INSA-CPE, Malaria Research Unit, – 43 boulevard du 11 novembre 1918 69622 Lyon France; 3 Univ Lyon, Université Claude Bernard Lyon 1, Institut de Chimie, de Biologie Moléculaire et Supramoléculaire ICBMS-UMR5246, CNRS-INSA-CPE, – 43 boulevard du 11 novembre 1918 69622 Lyon France

**Keywords:** malaria susceptibility, Nutrition, AGE, Fulani, Dogon, Mali

## Abstract

There are growing data supporting the differences in susceptibility to malaria described between sympatric populations with different lifestyles. Evidence has also been growing for some time that nutritional status and the host’s metabolism are part of the complex mechanisms underlying these differences. The role of dietary advanced glycation end-products (AGEs) in the modulation of immune responses (innate and adaptive responses) and chronic oxidative stress has been established. But less is known about AGE implication in naturally acquired immunity and susceptibility to malaria. Since inflammatory immune responses and oxidative events have been demonstrated as the hallmark of malaria infection, it seems crucial to investigate the role of AGE in susceptibility or resistance to malaria. This review provides new insight into the relationship between nutrition, metabolic disorders, and infections, and how this may influence the mechanisms of susceptibility or resistance to malaria in endemic areas.

## Abstract (fr)

De plus en plus de données étayent les différences de susceptibilité au paludisme décrites entre les populations sympatriques avec des modes de vie différents. Il existe également de plus en plus de preuves depuis un certain temps que l’état nutritionnel et les métabolismes de l’hôte font partie des mécanismes complexes qui sous-tendent ces différences. Le rôle des produits terminaux de glycation avancée d’origine alimentaire (AGE) dans la modulation des réponses immunitaires (réponses innée et adaptative) et le stress oxydatif chronique a été établi. Mais on connaît peu sur l’implication des AGE dans l’immunité naturellement acquise et la sensibilité au paludisme. Puisqu’il a été démontré que les réponses immunitaires de types inflammatoires et les événements oxydatifs sont caractéristiques de l’infection au paludisme, il semble primordial d’étudier le rôle des AGE dans la sensibilité de la résistance au paludisme. Cette synthèse fournit un nouvel aperçu sur la relation entre la nutrition, les maladies métaboliques et les infections, et comment cela peut influencer les mécanismes de susceptibilité ou de résistance au paludisme en zone d’endémie.

## 1. Introduction

Infectious diseases, such as malaria, are no longer considered as a random encounter between a microbe, particularly the eukaryotic parasite, and its vertebrate or human host. The outcome of this primary encounter is driven by many related factors. Virulence of the microbe, infectious burden, route of inoculation, the host’s immunity, susceptibility to infection, and previous drugs used, are among these well-documented factors. Compelling evidence has been growing for some time that the nutritional status of the host is part of this complex relationship. Parasitism has some specificity in terms of the host-pathogen relationship that confers selectivity for the host to the parasite (adaptation).

The nature, magnitude, and effectiveness of the immune response induced by the malaria parasite determines the clinical outcome of the current malaria episode, as well as the susceptibility to subsequent episodes [18, 41]. The complex interactions between environment, lifestyle, and the modulation of the host’s immune responses constitute a new area of great interest in malaria research. The role of the gut microbiota is now established in the natural resistance to malaria [82]. But less is known about the role of host metabolism in this natural resistance/susceptibility to malaria. In this regard, investigating the host diet and metabolism could provide a better understanding of the mechanisms involved in hosts’ natural defenses again pathogens.

Dietary advanced glycation end-products (AGEs) and their specific receptors (receptors of AGE: RAGE) constitute a link between the environment, lifestyle, and the immune system [1]. The role of AGEs has been investigated in the occurrence of chronic and inflammatory diseases [14, 61], as well as in susceptibility of patients to *Streptococcus* infection, peritonitis, and sepsis [17, 46]. Recently, the role of dietary AGE in both innate and adaptive immune response modulation was described. Despite the well-documented deleterious effects of AGE on immunity by shifting the Th1/Th2 balance toward an excessive Th1 response and perpetuating oxidative stress, no study has yet investigated their role in susceptibility or resistance to malaria.

The aim of this review is to present and discuss the potential role of diet intake advanced glycation end-products and their specific receptors in modulation of the immune response, and their implication in susceptibility to infection and clinical malaria.

### 1.1. Disease burden

Malaria remains one of the most challenging diseases in terms of mortality and drug resistance rates. The World Health Organization (WHO) recorded 198 million cases of malaria in 2013, with approximately 584,000 cases of death, of which 90% occurred in Africa [76]. Around 2,000 people (majority under age five) die per day from malaria. Malaria remains endemic in many tropical areas, particularly in Africa and Asia [74]. Despite many years of research on malaria vaccines, only one candidate reached phase III with an efficacy of ~50% [58], and resistance to antimalarial drugs is increasing. Mosquito resistance to insecticides and *Plasmodium* resistance to drugs, including the most recent artemisinin derivatives, are of great concern worldwide. The need to develop new strategies and explore new research avenues and hypotheses on biological factors of malaria susceptibility or resistances is becoming more and more relevant.

## 2. Advanced glycation end-products (AGEs) and their specific receptors (RAGE)

**Figure 1.**
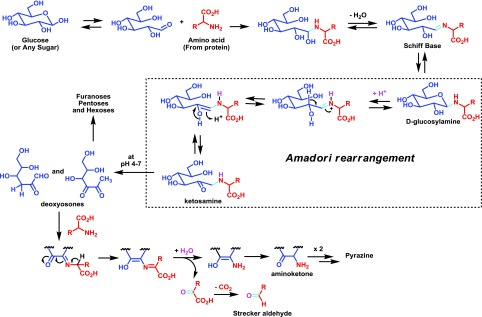
Major products resulting from the reaction of amino acids (from protein) and reducing sugars during the Maillard reaction.

The AGEs are derived from a non-enzymatic reaction (Maillard reaction, described by Louis Camille Maillard in 1912) [81]. The Maillard reaction links protein amino groups with reducing glucose-derived carbonyl groups; leading to the glycation of the protein, altering its structure and function (Fig. 1). The reaction occurs in several stages: an initial step involves the condensation of an amino acid (from a protein) and an opened form of a glucose-derived product (aldehyde-derived sugar), giving after loss of a water molecule, a Schiff base which is in equilibrium with its closed form, a *D*-glucosylamine. Upon protonation, this latter is then transformed, after ring opening, into a ketosamine. Such amines can be degraded at pH 4–7 to provide several carbonyl-derived sugars such as deoxyosones but also furanoses, pentoses, and hexoses; upon condensation with an amino acid, the very reactive deoxysones will yield an aminoketone intermediate, after hydrolysis of an imine intermediate, which can either combine to provide pyrazine-derived products or after loss of carbon dioxide deliver a Strecker aldehyde, as an irreversible advanced end-product (Fig. 1) [54, 60].

Advanced glycation end-products are generated endogenously when the blood sugar level is chronically high (during diabetic) or oxidative stress, as well as exogenously during the cooking process of meals [25, 54, 61]. The quantity of AGEs generated in the food is positively correlated to the cooking temperature [54, 59]. Any difference in lifestyle and dietary habits could have a considerable impact on exposure to dietary AGE [29, 52–64]. Accumulation of AGEs in blood, tissues, and organs results in chronic toxicity and cell signaling engagement, with deleterious effects on innate and adaptive immune defense [1, 52, 75]. *In vivo*, AGEs act as damage-associated molecular patterns (DAMPs) with their specific receptors known as receptors for advanced glycation end-products (RAGE).

The receptor for advanced glycation end-products (RAGE) is a transmembrane protein on the cellular surface that recognizes tridimensional molecules, instead of amino-acid sequences. RAGE contains two constant (C-type) and a variable (V-type) immunoglobulin-like domains. Different variants of RAGE exist, including the soluble one (sRAGE). sRAGE only has the extracellular domain of the receptor. sRAGE acts as a decoy receptor for AGEs and may prevent inflammation [78–80]. Other AGE-sensitive receptors have been described, including the oligosaccharyl transferase complex protein 48 (OST-48 or AGE receptor-1 (AGER1) involved in anti-oxidant mechanisms), 80-KH protein (AGER2 – AGE receptor-2), galectin-3 (AGER3 – AGE receptor-3), and some scavenger receptors. But RAGE is the most investigated of these and seems to be the most important in cell signaling pathways [28]. RAGE is expressed at high levels in the lungs and at low levels in normal adult tissues and cells involved in the innate immune system, e.g. neutrophils, T and B lymphocytes, monocytes, macrophages, dendritic cells, and endothelial cells [9, 16, 50]. In addition to AGE, RAGE can interact with diverse ligands such as: high-mobility group box 1 protein (HMGB1 or amphoterin), the group of calcium-binding cellular factors S100 (calgranulin), amyloid beta peptides, and Mac-1, a beta-2 integrin (CD1lb/CD18).

The activation of RAGE triggers a series of cellular signaling events, including the activation and translocation into the nucleus of transcription factor *NF-kappa B*, leading to the production of pro-inflammatory cytokines, chemokines, adhesion molecules, and oxidative stress. The recruitment of RAGE by AGE also leads to endothelial cell activation [7]. Upon binding with its ligands, RAGE is overexpressed and becomes part of a positive feedback-loop mechanism that results in auto-amplification of RAGE expression, chronic cell signaling, and oxidative stress [6]. The expression of RAGE on cells is therefore highly regulated by dietary AGEs [54, 78–80].

## 3. RAGE polymorphism, natural selection pressure, and immune system regulation

*Homo erectus* domesticated fire and started cooking meat about 400,000 years ago [20]. During this permanent exposure to dietary glycation end-products, *Homo sapiens* could have co-evolved with AGEs by developing genetic polymorphisms on the *RAGE* gene. The gene encoding RAGE is located on the major histocompatibility complex region that comprises the HLA gene loci [78]. Since the potential involvement of HLA-DRB1*04 and -DQB1*02 alleles in enhanced immune reactivity is documented, as well as their high frequency in the Fulani ethnic group [44], it seems important to consider the relationship between *RAGE* gene polymorphism and susceptibility to malaria in endemic areas.

The involvement of some genetic polymorphisms (single nucleotide polymorphism: SNP) on RAGE promoters in the susceptibility or resistance to some metabolic infectious diseases suggests that RAGE is sensitive to natural selective pressure. SNPs 374T/A and 429T/C in the RAGE promoter region are involved in the regulation of RAGE expression by acting on the nuclear protein-binding site [31]. This polymorphism may be involved in the modulation of immunity by hyper-expression of variants of anti-inflammatory genes of immunity [31]. A positive association between allele A of 374T/A polymorphism and longevity is described in the elderly population older than 90 years. Another SNP of the RAGE promoter (genotype 82SS) is associated with significantly elevated risk for cervical cancer (adjusted odds ratio = 1.98, *p* < 0.001); and significantly lower serum soluble RAGE (sRAGE) levels. In contrast, 82GS polymorphism is associated with higher serum sRAGE levels [77]. Higher levels of sRAGE are commonly described in healthy people. The role of sRAGE in host defense during severe infection is conflicting [47], but several experimental studies have reported a positive effect of sRAGE on the outcome of sepsis [10, 38].

The malaria parasite has co-evolved with human beings for about 100,000 years and the host has developed many protective strategies (HbS, HbC, HbE, G6PD, etc.) [39, 41]. While the human host has been perpetually under genetic and environmental selective pressure, the malaria parasite has also developed survival strategies to evade the host’s immune system and to become more adapted [41, 57, 87]. Some human malaria parasite species (*P. vivax, ovale*, *and malariae*) are more adapted to certain human subpopulations in endemic areas by developing host immune tolerance [57, 87], suggesting the interplay between environment, lifestyle, and immune responses [1]. Without treatment, *Homo sapiens* infection with *Plasmodium malariae* can stay lifelong in the host.

Differences in susceptibility to malaria are well established in sympatric populations with different lifestyles in sub-Saharan Africa [4, 13, 23]. Despite several investigations conducted in these populations, the biological and environmental factors involved in the mechanisms underlying this difference of susceptibility or resistance remain poorly understood [22, 23, 35–37, 45, 53]. One of the main differences between these populations is lifestyle. The nutritional status and metabolism of the host are determinants in the clinical outcome of malaria infection [8, 12, 19, 26, 32] by modulating the immune responses and oxidative stress [33, 34].

## 4. RAGE, AGE and infectious diseases

There is evidence that the interplay between some nutrient-derived metabolites (arylhydrocarbon, vitamins A and D) and their nuclear receptors plays a key role in maturation of the lymphoid organs and modulation of the immune system [66, 72]. The aryl hydrocarbon receptor (AhR), the retinoic acid receptor (RAR), and the vitamin D receptor (VDR) are expressed on immune system cells (TH17 and Treg cells, γδ T cells expressing IL-17, CD4+ T cells, TH17-derived TH1, and Tr1 cells) [56, 72]. Available data support the idea that these nuclear receptors are directly influenced by the environment and especially by the diet, with a considerable impact on development and specialization of the types of immune responses [49, 73].

RAGE is a cellular receptor that interacts highly with nutrient-derived metabolites. Growing evidence supports the relevance of the RAGE signaling pathway in the pathogenesis of many diseases like diabetes and its associated complications, but also in certain clinical settings. RAGE, as a pattern-recognition receptor, seems to have a crucial role in early host defense against invading pathogens by activating innate immune response, which primes acquired immunity, particularly by driving induction of a Th1-type response [27]. Many studies have revealed the role of RAGE in inflammatory cell recruitment, extravasations of leukocytes across the endothelial barrier with further inflammatory events, and induction of apoptosis [24, 42, 71, 83, 85, 86]. Martinez et al., in 2014, reported that RAGE was involved in the susceptibility of diabetic patients to *Streptococcus pneumoniae* infections by impaired CD4^+^ and Th17 cell memory responses [46]. Experimental studies provided further insight into the role of RAGE and its ligands in host defense during clinically important infections, suggesting RAGE as a potential target for therapeutic strategies [51]. Inhibition of RAGE signaling reduces inflammatory responses in infectious models of diseases [70]. Blockade of interactions between RAGE and its ligands results in improved outcome from sepsis [69] and *influenza A* virus pneumonia [68]. However, some conflicting data indicate increased bacterial growth in experimental models as the result of inhibition of RAGE engagement [67]. The fact that the effect of RAGE during infection could differ whether the pathogen is Gram positive (deleterious effect) or negative (beneficial effect), also suggests that RAGE could interact with ligands from the pathogen [71]. The apparent discrepancy between the effects of RAGE in experimental infection could result from this specific host-pathogen interaction.

The innate immune response induced by RAGE when activated by AGE is a sterile immune reaction. The sterile inflammatory immune response is similar to infection immune response, including the recruitment of neutrophils and macrophages, the production of inflammatory cytokines and chemokines, and the induction of T cell-mediated immune responses [15]. The magnitudes of innate as well as the type of adaptive immune responses are strongly influenced by the cellular expression of RAGE in immune effector cells [1]. *T helper* 2 (*Th2*) cytokine production, which is frequently downregulated in T cells expressing RAGE, is critical for host defense against blood stage malaria [55].

The role of RAGE-AGE in the susceptibility or resistance to malaria remains unknown and needs to be investigated. Increased susceptibility to clinical malaria has already been described in 2-year-old children with higher plasma levels of advanced oxidative protein products (AOPP), compared to those with lower plasma AOPP [84].

But no study has directly investigated the relationship between AGE-RAGE signaling and malaria susceptibility. During sepsis, high-mobility group box-1 (HMGB1), a DNA- and heparin-binding protein with pro-inflammatory activity, enhances and prolongs inflammatory processes. HMGB1 is one of the ligands of RAGE and shares part of the RAGE-AGE pathway. More than a decade ago, HMGB1 was shown to be increased in *falciparum* malaria and was suspected of being an amplification signal in disease pathogenesis, and thus illness [2]. Furthermore, HMGB1 has been suggested as an informative prognostic marker of disease severity in severe human malaria [3, 30]. A DNA-binding protein also described in *P. falciparum* (Pf-HMGB1/2), which has 98% similarity with human HMGB1, has been found to be able to activate RAGE and induce secretion of pro-inflammatory cytokines by human immune cells [40].

**Figure 2.**
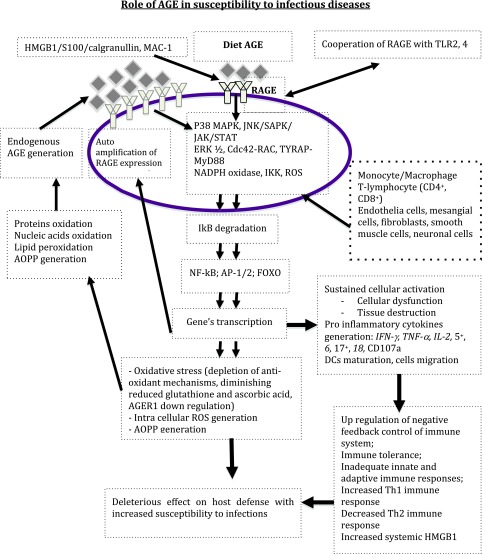
Role of diet AGE in perpetuating oxidant stress and pro inflammatory cytokines production with deleterious effect on immunity. *Abbreviation*: AGE: Advanced glycation endproducts; Cdc42-Rac: cell division control protein 42 homolog-Rac; ERK1/2: extracellular signal-regulated kinase ½; FOXO: forkhead box protein O subclass; HMGB1: High mobility group box 1; IkB: Inhibitor of Nuclear factor B; IKK: Inhibitor of nuclear factor B kinase; JAK: Janus kinase; JNK: c-jun N-terminal kinase;  MAC-1: macrophage-1 antigen; NADPH: nicotinamide adenine dinucleotide phosphate;  NF kB: Nuclear factor kapa B; P38MAPK: Mitogen associated protein kinase P38; RAGE: receptor for Advanced glycation endproducts; ROS: reactive oxygen species; SAPK: stress-activated protein kinase; STAT: signal transducer and activator of transcription; TLR: Toll-Like Receptor.

Malaria is endemic in some countries and sometimes leads to genetic diversity (blood group polymorphism) in endemic areas by positive selection. We hypothesize that in malaria endemic areas where populations are exposed to high levels of AGE in food, the chronic activation of RAGE by dietary AGE could lead to (Fig. 2):

- Chronic inflammatory immune response that results in immune tolerance (by upregulation of negative feedback control), cell dysfunction, tissue destruction, and exhaustive immune cells.
- Depletion of Th2 immune response and impairment of the Th1/Th2 immune response balance, with deleterious effects on the host-pathogen interaction.
- Chronic production of elevated HMGB1, which could recruit new RAGE and maintain a vicious circle of “RAGE overexpression → AGE/HMGB1 production → RAGE activation and overexpression”.
- Chronic oxidative stress with generation of reactive oxygen species (ROS), oxidation and glycation of proteins, nucleic acids, and lipids; with auto-amplification of AGE and AOPP generation.
- Selection of SNPs on the gene coding for RAGE with protective effects against malaria.

## 5. Food intake differences between ethnic groups and malaria susceptibility in endemic areas

Natural resistance to malaria has been described among pastoral populations (Fulani) compared to their neighboring farmers (Dogon) in Mali, or Mossi in Burkina Faso [5, 64]. Fulani populations who have approximately 50% lactose tolerance (milk constitutes their main meal) live in sympatric conditions with Dogon populations in the same environment in Mali [11, 21, 65], Fulani are less susceptible to clinical malaria and exhibited higher baseline levels of inflammatory cytokines (IL-6, IL-8, IL-12p70, IFN-α, IFN-γ) as well as malaria-specific antibodies (IgG, IgG1-3, and IgM) compared to Dogon [5, 11, 21, 65]. Except for IFN-γ that significantly increased in infected Fulani children, the inflammatory response was not influenced by active malaria infection. In the same populations, Arama et al. described an inhibition of toll-like receptor (TLR) response and altered antigen-presenting cell activation (dendritic cells) in Dogon, in contrast with their neighbors Fulani who exhibited an activation of dendritic cells and no altered innate immune defense during *Plasmodium falciparum* infection [4].

These populations living in malaria endemic areas are exposed to the same risk of malaria infection and disease. The main difference between the two population groups is their dietary habits. While Fulani have milk and couscous as their main food, Dogon have at least three well-cooked meals a day. Cooked foods contain more AGEs than milk and couscous. Even though the protective role of diet against malaria in Fulani has not yet been thoroughly investigated [43], these observations suggest that nutrition and metabolism could play a key role in malaria susceptibility/resistance in endemic area. The modulation of the types of immune response (Th1/Th2) is sensitive to the host’s diet. Deficiency of some nutrients can influence the balance of immune response [48].

It is well documented in the literature that reduction of dietary AGE consumption protects against loss of innate defense [75]. There is a correlation between low diet AGE intake and high cell expression of AGE receptor-1 (AGER1), which is an anti-oxidant receptor involved in AGE endocytosis and degradation, also contributing to protection against impairment of innate responses and oxidative stress [54].

More studies comparing the serum levels of AGE, sRAGE, RAGE expression, and RAGE polymorphism in these populations could provide new insight into the mechanism of malaria susceptibility or resistance.

## 6. Conclusion

In malaria endemic areas, people are exposed to multiple malaria infections. This exposure leads to the development of innate and adaptive immune responses against malaria, and genetic polymorphism in the long term.

In populations with AGE-rich cooked foods as their main food source, chronic activation of RAGE by AGE may induce and maintain pro-inflammatory cytokine production, oxidative stress, reactive oxygen species (ROS), and AOPP production, and result in innate immune tolerance, inadequate adaptive immune response, cellular dysfunction, and depletion of anti-oxidative mechanisms. The disturbance of host defense systems may increase susceptibility to malaria in these populations.

Targeting the dietary AGE-RAGE signaling pathway as a new research perspective in populations with different lifestyles could contribute to understanding the mechanism of the differences of susceptibility to malaria observed in some African populations. Such new strategies could contribute to reducing morbidity and mortality related to malaria in endemic countries by acting on lifestyles of populations, as already implemented in the control of some chronic metabolic diseases like diabetes and hypertension. The determination of blood levels of AGE, sRAGE, and cell expression of RAGE and AGER1, as well as polymorphism of genes encoding for these receptors, will provide relevant information on malaria susceptibility.

## Competing interests

The authors declare no competing interest.
